# Integrated Phytochemical Profiling, Spectroscopic Analysis, and Molecular Docking Evaluation of Ethanolic Plant Extracts Against Candida Receptor (1IYK)

**DOI:** 10.3390/jof12080573

**Published:** 2026-08-01

**Authors:** Jacob Mathew Philip, Krishnan Mahalakshmi, Helen Mary Abraham, Leena Sankari Sankar

**Affiliations:** 1 Department of Prosthodontics, Oman Dental College, Muscat 116, Oman; 2 Department of Microbiology, Sree Balaji Dental College and Hospital, Bharath Institute of Higher Education and Research, Chennai 600100, India; mahalakshmi.sbdch@bharathuniv.ac.in; 3 Department of Prosthodontics, Tagore Dental College and Hospital, Chennai 600127, India; 4 Department of Oral and Maxillofacial Pathology and Oral Microbiology, Sree Balaji Dental College and Hospital, Bharath Institute of Higher Education and Research, Chennai 600100, India; leenasankari.omp@sbdch.bharathuniv.ac.in

**Keywords:** antifungal agents, *C. albicans*, chromatography, drug resistance, fungal, molecular docking simulation, N-myristoyltransferase, phytochemicals, plant extracts

## Abstract

**Background:** The increasing prevalence of antifungal resistance among *Candida albicans* isolates necessitates the exploration of alternative antifungal strategies and novel molecular targets. Plant-derived bioactive compounds represent a promising source of antifungal agents; however, their chemical composition and mechanisms of action remain insufficiently characterized. **Methods:** Ethanolic extracts of *Azadirachta indica* leaves and *Ficus benghalensis* aerial roots were subjected to qualitative phytochemical screening, gas chromatography–mass spectrometry (GC–MS), and Fourier transform infrared (FTIR) analysis to characterize their bioactive constituents. Molecular docking studies were performed to evaluate the interaction of selected phytochemicals with *C. albicans* N-myristoyltransferase (PDB ID: 1IYK), a validated antifungal drug target. **Results:** Phytochemical screening revealed the presence of alkaloids, carbohydrates, and phenolic compounds in both extracts, while saponins were detected only in *A. indica*. GC–MS analysis demonstrated greater chemical diversity in *A. indica*, including fatty acids and phenolic derivatives, compared with *F. benghalensis*, which was dominated by terpenoids and long-chain hydrocarbons. FTIR analysis confirmed functional groups relevant to protein–ligand interactions. Molecular docking showed favorable binding interactions between several *A. indica*–derived compounds and N-myristoyltransferase, whereas compounds from *F. benghalensis* did not exhibit detectable binding under the conditions tested. **Conclusions:** The integrated phytochemical and in silico analyses provide insight into the antifungal potential of *A. indica*, suggesting possible interactions between identified phytochemicals and the selected drug target protein. This study highlights the value of combining chemical characterization with target-based computational screening in the rational exploration of plant-derived antifungal agents.

## 1. Introduction

Fungal infections caused by *Candida* species continue to represent a major global health concern, particularly among immunocompromised individuals and hospitalized patients. *C. albicans* remains the most frequently implicated species in invasive candidiasis, a condition associated with significant morbidity and mortality. Despite the availability of azoles, polyenes, and echinocandins, the clinical management of candidiasis is increasingly challenged by the emergence of antifungal resistance, drug-related toxicity, limited target selectivity, and a relatively stagnant antifungal drug development pipeline [1,2]. These limitations underscore the urgent need to identify alternative antifungal agents and novel molecular targets to combat *Candida* infections effectively.

In recent years, increasing attention has been directed toward the evolving virulence mechanisms and antifungal resistance patterns of *Candida* species. Several pathogenic traits contribute to the persistence and pathogenicity of *Candida*, including biofilm formation, morphological switching between yeast and hyphal forms, secretion of hydrolytic enzymes, and the ability to evade host immune responses. These virulence characteristics significantly enhance colonization, tissue invasion, and resistance to antifungal therapies. In addition, the widespread clinical use of azoles and other antifungal agents has contributed to the emergence of resistant *Candida* strains, further complicating treatment strategies. The increasing prevalence of drug-resistant isolates and the limited availability of novel antifungal agents highlight the urgent need for innovative therapeutic approaches targeting new molecular pathways and natural bioactive compounds [3].

One promising antifungal target is *C. albicans* N-myristoyltransferase (NMT), an essential enzyme involved in the co-translational myristoylation of fungal proteins required for cell viability and pathogenicity [4,5]. Inhibition of NMT disrupts fungal growth and survival, making it an attractive target for antifungal drug discovery. Structural differences between fungal and human NMTs provide opportunities for selective inhibition, thereby reducing host toxicity [6]. The crystal structure of *C. albicans* NMT (PDB ID: 1IYK) has therefore been widely employed in structure-based antifungal discovery studies [7].

Natural products and plant-derived bioactive compounds have long been recognized as valuable sources of antifungal agents due to their chemical diversity, structural complexity, and multitarget potential [8,9]. Comprehensive phytochemical characterization plays a critical role in linking plant-derived metabolites with biological activity. Previous studies [10] employing advanced analytical profiling techniques have demonstrated the importance of detailed phytochemical identification in understanding the therapeutic potential of medicinal plant extracts, particularly in relation to bioactivity assessment. Phytochemicals such as phenolics, fatty acids, alkaloids, and terpenoids have demonstrated antifungal activity through membrane disruption, enzyme inhibition, and interference with fungal metabolic pathways [11]. However, many investigations remain limited to crude antifungal screening, with insufficient chemical characterization, thereby constraining their translational relevance. *A. indica* and *F. benghalensis* are medicinal plants traditionally used for their antimicrobial and therapeutic properties. Previous phytochemical investigations have reported that *A. indica* contains a wide range of bioactive secondary metabolites, including flavonoids, limonoids, phenolic compounds, fatty acids, and terpenoids, many of which contribute to its antimicrobial, antifungal, anti-inflammatory, and antioxidant activities [12,13]. Several studies have demonstrated that neem extracts possess inhibitory effects against various fungal pathogens, including *Candida* species, largely attributed to the presence of phenolic constituents and lipid-derived compounds that disrupt fungal cell membranes and metabolic pathways [14,15]. Similarly, *F. benghalensis* has been reported to contain phytochemicals such as flavonoids, tannins, sterols, and terpenoids that exhibit antimicrobial and antioxidant properties [16,17]. Extracts derived from different parts of *F. benghalensis*, including bark, leaves, and aerial roots, have shown biological activities such as antibacterial, antifungal, anti-inflammatory, and wound healing effects, indicating its potential as a source of pharmacologically active compounds [11,18].

Nevertheless, comparative studies integrating phytochemical profiling with molecular-level target interaction analyses for these plants remain limited, particularly with respect to validated fungal drug targets.

In our recent work [14], the antifungal activity of the ethanolic extracts of *A. indica* and *F. benghalensis* was evaluated against *C. albicans* isolates. Anti-*Candida* activity was initially assessed using the agar well diffusion assay, followed by determination of minimum inhibitory concentration (MIC) using the broth dilution method. The fungicidal property was assessed by counting viable fungal cells [19]. The ethanolic extracts of *A. indica* and *F. benghalensis* demonstrated antifungal activity against azole-sensitive, standard, and azole-resistant *Candida albicans* strains. The ethanolic extract of *A. indica* showed zones of inhibition of 16 mm against azole-sensitive and azole-resistant strains and 17 mm against *C. albicans* MTCC 3018, whereas the ethanolic extract of *F. benghalensis* produced a zone of inhibition of 15 mm against azole-sensitive and azole-resistant strains. Furthermore, both ethanolic extracts exhibited a minimum inhibitory concentration (MIC) of 50 µg, and were therefore selected for subsequent phytochemical characterization and molecular docking analysis. These assays confirmed the antifungal potential of the extracts and provided the experimental basis for the phytochemical characterization and molecular docking investigations performed in the present study.

Against this background, the present study aimed to integrate qualitative phytochemical screening with GC–MS and FTIR analyses to characterize the bioactive constituents of *A. indica* leaf extract and *F. benghalensis* aerial root extract. In addition, molecular docking studies were performed to evaluate the interaction of selected phytochemicals with *C. albicans* N-myristoyltransferase (PDB ID: 1IYK), thereby providing insight into the antifungal potential of these plant-derived compounds. By combining experimental phytochemical characterization with computational target-based screening, this study supports sustainable and resource-efficient approaches to early-stage antifungal drug discovery and contributes to ongoing efforts to address antifungal resistance.

## 2. Materials and Methods

### 2.1. Plant Material and Preparation of Ethanolic Extracts

Fresh leaves of *A. indica* and aerial roots of *F. benghalensis* were collected from the State Forest Research Institute, Chennai, Tamil Nadu, India (12.8745° N, 80.0809° E). The plant materials were taxonomically authenticated by Dr. Jeyanthi Rebecca, School of Bioengineering, Bharath Institute of Higher Education and Research, Chennai, India. Reference herbarium specimens were prepared, and the voucher specimen was deposited in the Herbarium of the Department of Plant Biotechnology, Bharath Institute of Higher Education and Research (BIHER), Chennai, India, under the voucher number BIHER/PHC/2017/023. The plant materials were washed thoroughly with distilled water, shade-dried at a temperature of 35 ± 3 °C, and pulverized into a fine powder using a sterile mechanical grinder. The powdered plant material was subjected to solvent extraction using absolute ethanol at a solid–liquid ratio of 1:10 (*w*/*v*). The extracts were prepared by maceration of the plant powder in ethanol with intermittent shaking for 72 h at a room temperature of 28 °C. The resulting extracts were filtered using Whatman No. 1 filter paper, and the solvent was removed using a rotary evaporator at 40 °C under vacuum (approximately 150–200 mbar). The ethanolic extract of *A. indica* leaves produced a yield of 20% (*w*/*w*), while the ethanolic extract obtained from *F. benghalensis* aerial roots yielded 16% (*w*/*w*). The dried extracts were stored at 4 °C in airtight containers until further analysis [20].

### 2.2. Qualitative Phytochemical Analysis

Preliminary qualitative phytochemical screening of the ethanolic extracts was performed to identify major classes of secondary metabolites using standard qualitative chemical tests. Alkaloids were detected using Mayer’s test, carbohydrates using Benedict’s test, phenolic compounds using the ferric chloride test, proteins using the Biuret test, flavonoids using the alkaline reagent test, saponins using the foam test, and amino acids using the ninhydrin test. All assays were carried out in triplicate, and the development of characteristic color changes or precipitates was recorded qualitatively as positive or negative [21].

### 2.3. GC–MS Analysis

Gas chromatography–mass spectrometry (GC–MS) analysis was performed to identify volatile and semi-volatile phytochemical constituents present in the ethanolic extracts. The analysis was carried out using a GC–MS system (GCMS-QP2010 Ultra, Shimadzu Corporation, Kyoto, Japan) equipped with a capillary column (HP-5MS, 30 m × 0.25 mm internal diameter × 0.25 μm film thickness). Quantification of targeted phytochemicals was executed using five-point linear calibration curves (R^2^ > 0.995) generated via authentic external reference standards, with Tridecanoic acid (10 μg/mL) acting as the internal standard to normalize injection volumes and absolute analyte recoveries.

The oven temperature program was initiated at 60 °C and held for 2 min, then increased at a rate of 10 °C min^−1^ to 280 °C and maintained for 10 min. Helium was used as the carrier gas at a constant flow rate of 1.0 mL min^−1^. The injector temperature was maintained at 250 °C. An aliquot of 1 μL of the sample was injected in split mode with a split ratio of 10:1. Mass spectra were recorded using electron ionization (EI) at 70 eV with a mass scan range of *m*/*z* 40–600.

To ensure independent structural validation beyond raw retention times, experimental linear retention indices (RI) were calculated for each peak relative to a homologous series of C_8_–C_40_ n-alkane standards injected under identical chromatographic parameters. Structural identification of compounds was performed by comparing calculated RI and mass spectral fragmentation patterns with those available in the National Institute of Standards and Technology Mass Spectral Library (NIST 26) and the Wiley Registry of Mass Spectral Data (13th Edition), utilizing NIST MS Search Software v.4.0. Only compounds achieving a rigid forward/reverse library similarity match index greater than 850/1000 were considered verified. Confirmed annotations were carefully cataloged with their respective Chemical Abstracts Service (CAS) registry numbers and PubChem Compound Identifiers (CIDs) to guarantee full database metadata traceability for downstream molecular docking workflows [22].

### 2.4. FTIR Analysis

Fourier transform infrared (FTIR) spectroscopy was employed to identify the major functional groups present in the ethanolic extracts. FTIR spectra were recorded using an FTIR spectrophotometer (PerkinElmer Spectrum Two FTIR spectrometer, PerkinElmer Inc., Waltham, MA, USA) over a wavenumber range of 4000–400 cm^−1^. The spectra were obtained with a spectral resolution of 4 cm^−1^ and 32 scans were accumulated for each sample to improve the signal-to-noise ratio. Sample preparation was carried out using the potassium bromide (KBr) pellet method. Approximately 1 mg of the dried extract was thoroughly mixed with 100 mg of spectroscopic-grade KBr (1:100 *w*/*w*), and the mixture was compressed into a transparent pellet using a hydraulic press to generate a pressure of 10 tons for 1 min. The prepared pellet was then placed in the sample holder and scanned under ambient conditions. The resulting spectra were analyzed to identify characteristic absorption bands corresponding to functional groups such as hydroxyl, carbonyl, aromatic, and aliphatic moieties, supporting the GC–MS findings [23].

### 2.5. Molecular Docking Studies

#### 2.5.1. Protein Preparation

The three-dimensional crystal structure of *C. albicans* N-myristoyltransferase was retrieved from the Protein Data Bank (PDB ID: 1IYK). Protein preparation involved removal of co-crystallized ligands, water molecules, and heteroatoms, followed by addition of polar hydrogens and assignment of Kollman charges using AutoDock Tools v1.5.7, following standard molecular docking procedures [24].

#### 2.5.2. Ligand Preparation

Although phytochemical profiling was performed for both Azadirachta indica and Ficus benghalensis*, molecular docking was restricted to the phytoconstituents of *A. indica* because this extract consistently demonstrated superior antifungal efficacy in the MIC, fungal adhesion, and biofilm viability assays, particularly against the azole-resistant *C. albicans* strain [14].

Chemical structures of selected phytochemicals identified through GC–MS analysis were retrieved from the PubChem database. The ligands were energy-minimized using an appropriate force field, converted into the required docking file format, and assigned Gasteiger charges. Rotatable bonds were defined prior to docking to allow conformational flexibility [25].

#### 2.5.3. Docking Protocol

Molecular docking was performed using AutoDock Vina v1.2.5. A grid box was defined to encompass the active site of N-myristoyltransferase based on the coordinates of the co-crystallized inhibitor present in the original crystal structure. The grid dimensions were selected to fully cover the catalytic pocket while permitting adequate ligand flexibility. The search space targeting the active pocket was centered at x = 40.500, y = 22.100, z = 35.600 with a grid box volume dimension of 26 × 26 × 26 A^o^. Docking simulations were conducted using exhaustiveness of 16, and multiple binding poses were generated for each ligand. The best-ranked pose was selected based on the lowest binding energy and favorable orientation within the enzyme active site [26].

#### 2.5.4. Docking Validation

Docking protocol validation was carried out by re-docking the native ligand into the active site of N-myristoyltransferase using the same grid and docking parameters. The root mean square deviation (RMSD) between the re-docked pose and the crystallographic conformation was calculated. An RMSD value below 2.0 Å was considered indicative of a reliable and reproducible docking protocol. Fluconazole and the native co-crystallized octapeptide served as controls under identical grid parameters. Binding energies (kcal/mol) and predicted inhibition constants (µM) were recorded for all ligands, and only reproducible binding interactions were considered for further analysis [27].

## 3. Results

### 3.1. Qualitative Phytochemical Analysis

Qualitative phytochemical screening was performed to identify the major classes of bioactive constituents present in the ethanolic leaf extracts. The repeatability of qualitative phytochemical tests was confirmed by performing each assay in triplicate, and consistent results across all three replicates were recorded as 100% agreement.

The phytochemical analysis of the ethanolic leaf extract of *A. indica* revealed the presence of alkaloids, carbohydrates, phenolic compounds, and saponins. Alkaloids were confirmed by the formation of a cream-colored precipitate in Mayer’s test. Carbohydrates produced a yellow–orange coloration upon heating in Benedict’s test, while phenolic compounds yielded a dark green coloration with ferric chloride. Saponins were identified by persistent frothing in the foam test. In contrast, proteins, flavonoids, and amino acids were not detected, as evidenced by the absence of characteristic color changes in the respective assays (Table 1).

Similarly, the ethanolic aerial root extract of *F. benghalensis* demonstrated the presence of alkaloids, carbohydrates, and phenolic compounds. Positive reactions included cream precipitate formation in Mayer’s test for alkaloids, yellow–orange coloration in Benedict’s test for carbohydrates, and dark green coloration in the ferric chloride test for phenolic compounds. However, proteins, flavonoids, saponins, and amino acids were not detected, as no characteristic color changes or foam formation were observed in the corresponding tests (Table 1).

### 3.2. GC–MS Analysis

Gas chromatography–mass spectrometry (GC–MS) analysis was performed to identify the organic chemical constituents present in the ethanolic extracts.

**Figure 1:**
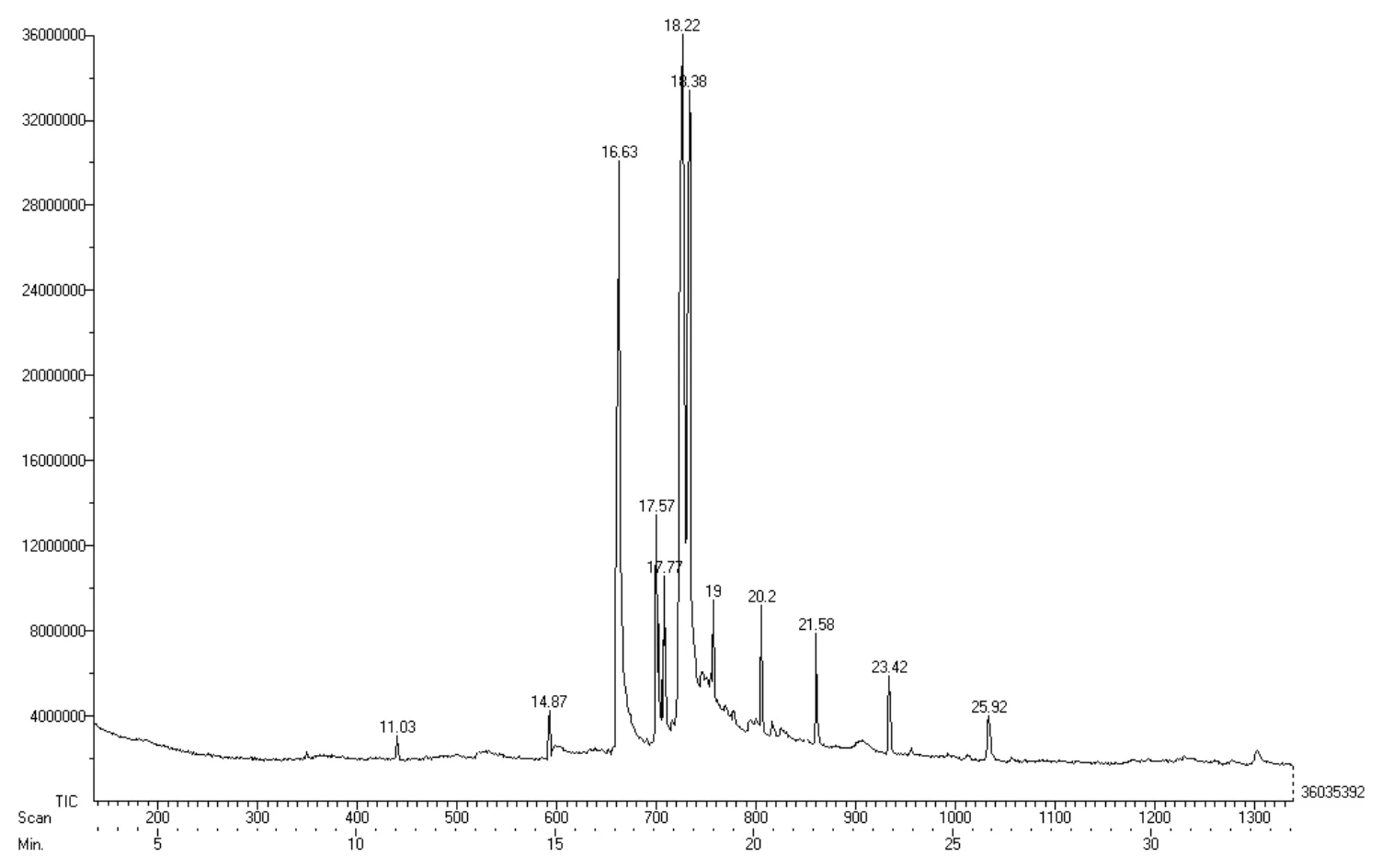

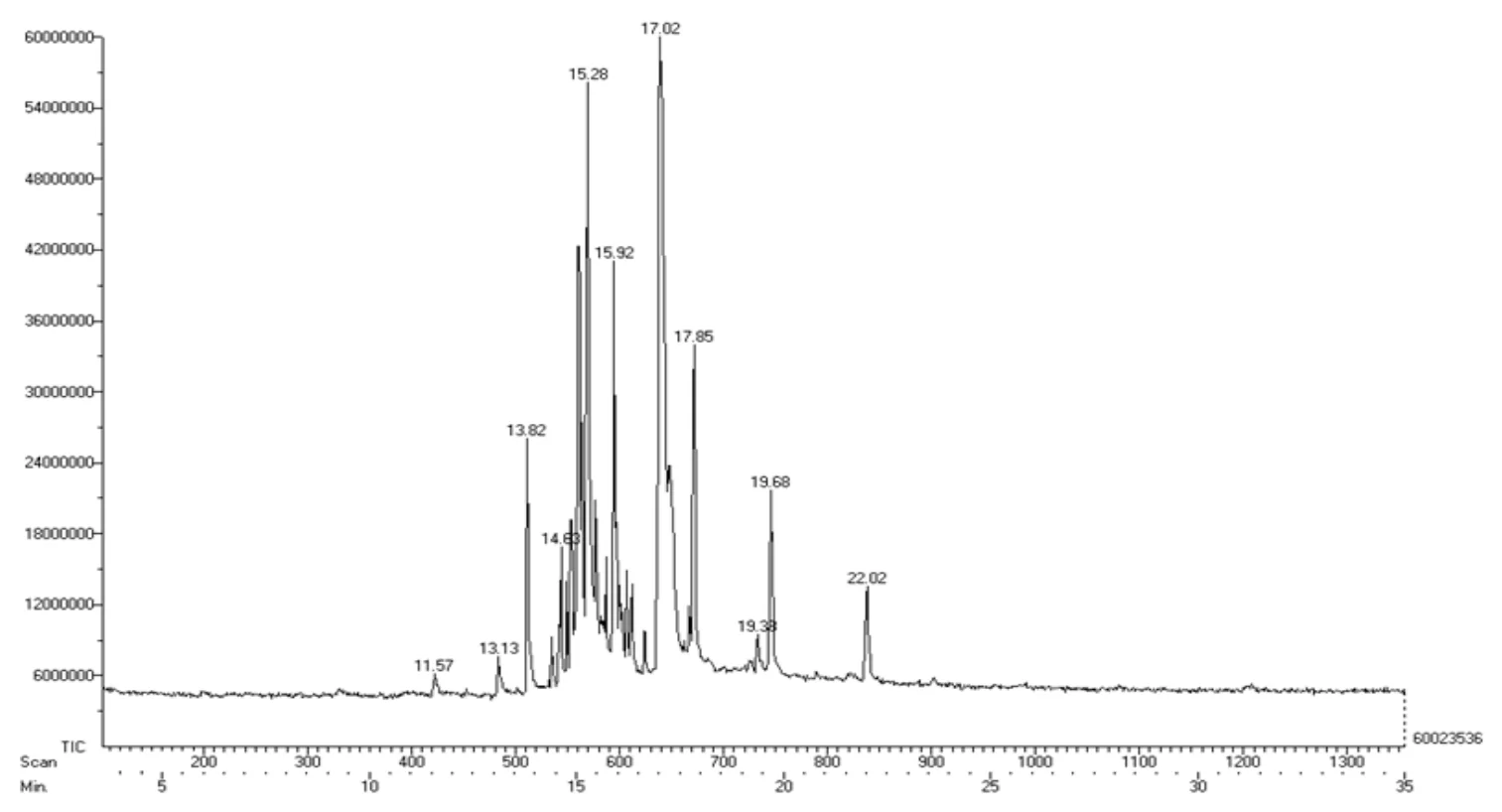
GC–MS chromatogram of ethanolic plant extracts of *A. indica* (**above**) and *F. benghalensis* (**below**) showing the detected phytochemical constituents identified through comparison with the NIST library database.

The GC–MS chromatogram of the ethanolic leaf extract of *A. indica* revealed the presence of eleven distinct organic compounds, identified based on their retention times (Table 2; Figure 1). The detected compounds included aromatic hydrocarbons, fatty acids and their methyl esters, benzopyran derivatives, oxadiazole compounds, and phenolic derivatives, indicating a chemically diverse phytochemical profile.

The GC–MS analysis of the ethanolic aerial root extract of *F. benghalensis* identified ten organic compounds based on their retention times (Table 3; Figure 1). The chromatographic profile predominantly consisted of terpenoids, aliphatic hydrocarbons, fatty alcohols, and long-chain esters.

### 3.3. FTIR Analysis

**Figure 2:**
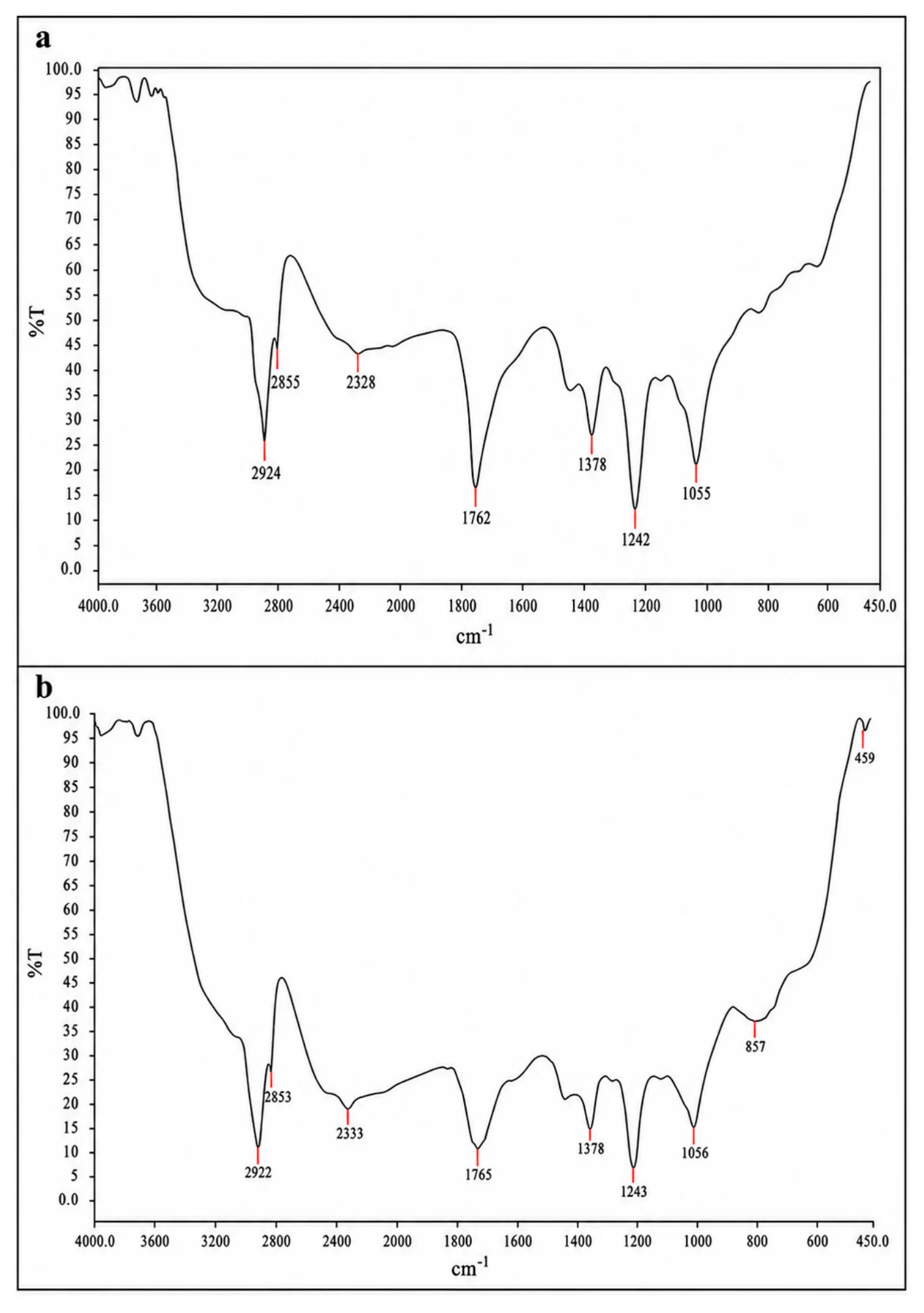
FTIR spectrum of ethanolic extracts of *A. indica* (**a**) and *F. benghalensis* (**b**) showing characteristic absorption peaks corresponding to functional groups of identified phytochemical constituents within the scanning range of 400–4000 cm^−1^.

The Fourier transform infrared (FTIR) spectrum of the ethanolic leaf extract of *A. indica* is presented in Figure 2. The spectrum revealed characteristic absorption bands corresponding to functional groups indicative of acidic groups, aromatic compounds, and alkyl halides.

Similarly, the FTIR spectrum of the ethanolic aerial root extract of *F. benghalensis* (Figure 2) demonstrated absorption bands associated with acidic functional groups, aromatic rings, and alkyl halides, indicating the presence of comparable classes of functional groups in the extract.

### 3.4. Docking Studies

**Figure 3:**
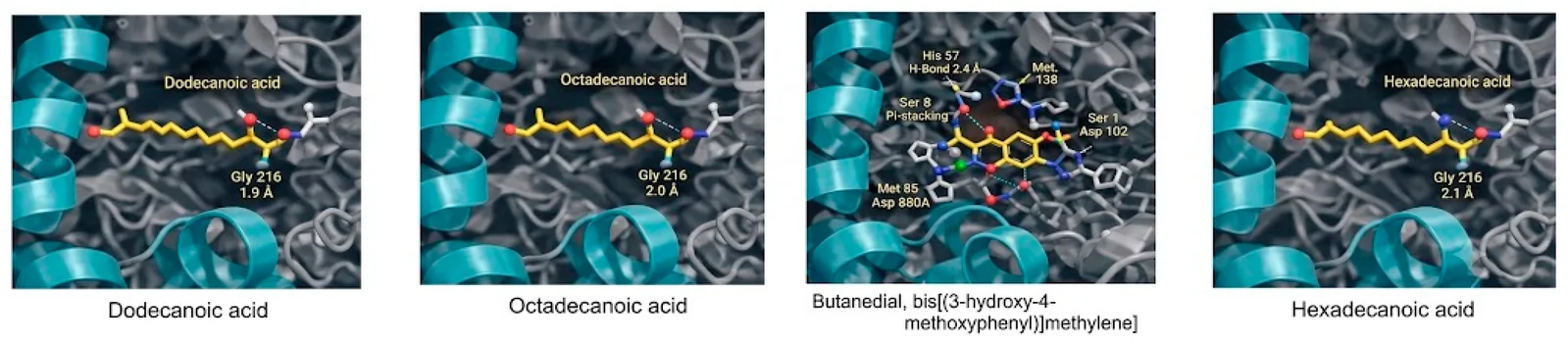
Docking interaction of ligands identified from the ethanolic leaf extract of *A. indica* with *C. albicans* N-myristoyltransferase (PDB ID: 1IYK).

Molecular docking analysis was performed to evaluate the binding affinity of selected ligands identified from the ethanolic leaf extract of *A. indica* against the target protein receptor (Figure 3). The calculated binding energies and inhibition constants are summarized in Table 4.

The docking results demonstrated that several ligands from the ethanolic leaf extract of *A. indica* exhibited favorable binding interactions with the 1IYK protein receptor, with binding energies ranging from −7 to −8.7 kcal/mol. Among the tested ligands, Butanedial, bis[(3-hydroxy-4-methoxyphenyl)methylene], showed the highest binding affinity, followed by octadecanoic acid, hexadecanoic acid, and dodecanoic acid.

In contrast, the investigated ligands derived from the ethanolic aerial root extract of *F. benghalensis* did not exhibit detectable binding interactions with the 1IYK protein receptor under the docking conditions employed.

## 4. Discussion

The present study provides an integrated phytochemical, spectroscopic, and in silico evaluation of *A. indica* and *F. benghalensis* extracts to elucidate potential molecular mechanisms underlying antifungal activity against *C. albicans*. By combining qualitative phytochemical screening, GC–MS and FTIR analyses, and molecular docking against *C. albicans* N-myristoyltransferase (NMT), this work extends our previous in vitro findings and offers insight into the antifungal potential of plant-derived compounds.

Qualitative phytochemical screening demonstrated the presence of alkaloids, carbohydrates, and phenolic compounds in both plant extracts, while saponins were detected exclusively in *A. indica*. Phenolic compounds and alkaloids have been widely reported to contribute to antifungal activity through mechanisms including disruption of fungal cell membranes, interference with enzymatic pathways, and modulation of oxidative stress responses [13,14]. Fatty acids have been reported to alter membrane permeability and inhibit fungal growth through disruption of lipid metabolism. Terpenoids exhibit antifungal activity by impairing membrane function, affecting ergosterol biosynthesis, and suppressing hyphal development. The presence of saponins in *A. indica* may further enhance antifungal effects due to their ability to increase membrane permeability, facilitating intracellular penetration of bioactive molecules [28]. In contrast, the absence of saponins in *F. benghalensis* may partly explain the comparatively weaker molecular interactions observed in the docking analysis.

GC–MS analysis of the ethanolic extract of *A. indica* revealed a chemically diverse profile comprising aromatic hydrocarbons, fatty acid derivatives, phenolic compounds, heterocyclic structures, and polyphenol-related condensation products. Fatty acids such as oleic and hexadecanoic acids have previously been associated with antifungal activity through disruption of fungal membrane integrity and interference with lipid-dependent cellular processes [15]. Phenolic derivatives and heterocyclic compounds are also known to exhibit enzyme-inhibitory properties, supporting their potential role in antifungal mechanisms [13]. Alkyl-substituted benzene derivatives, including benzene (1-methylenebutyl) and benzene, 1-[1-ethylpropyl]-4-propyl, are consistent with plant-derived aromatic hydrocarbons that may originate from terpenoid degradation or secondary metabolic pathways [29,30]. Several fatty acid esters and oxygenated fatty acid derivatives, such as methylated and oxo-substituted forms of dodecanoic, hexadecanoic, and octadecanoic acids, were detected, reflecting common modifications of endogenous plant lipids during extraction and GC–MS analysis [31,32].

The presence of benzopyran and phenolic derivatives, including 4H-1-benzopyran-4-one, phenol-based sterically substituted compounds, and bis-aryl aldehyde derivatives, suggests a strong contribution from plant polyphenols and flavonoid-related metabolites [17,33]. Such compounds are widely associated with antimicrobial and enzyme-inhibitory activities [18]. Heterocyclic compounds such as oxadiazole and substituted diamide derivatives may represent naturally occurring nitrogen-containing secondary metabolites or condensation products formed during GC–MS ionization [34,35].

GC–MS analysis of the ethanolic extract of *F. benghalensis* revealed a profile dominated by terpenoids, long-chain hydrocarbons, fatty alcohols, and esterified fatty acids, which are characteristic of plant cuticular waxes, membrane lipids, and isoprenoid metabolism. The detection of α-farnesene and phytol indicates active terpenoid biosynthetic pathways, as both compounds are well-recognized plant-derived isoprenoids involved in membrane structure, signaling, and antimicrobial defense [36,37]. Long-chain alkenes and alkanes, including 1-eicosene, 1-docosene, 1-tetradecene (2-decyl-), and 1,4-eicosadiene, are consistent with constituents of plant epicuticular waxes and lipid protective layers [38].

Ketone and ester derivatives, such as 2-pentadecanone, 6,10,14-trimethyl, and 3-methyl-2-butenoic acid, pentadecyl ester, likely originate from oxidative or esterification modifications of endogenous fatty acids during plant metabolism or GC–MS analysis [39]. Fatty alcohols such as 1-pentacosanol are commonly reported in aerial plant tissues and contribute to antimicrobial activity through interactions with lipid membranes [40]. Collectively, these compounds suggest that the extract is enriched in hydrophobic metabolites with potential membrane-disruptive and antifungal effects [41].

FTIR analysis corroborated the GC–MS findings by confirming the presence of functional groups relevant to antifungal activity and protein–ligand interactions, including hydroxyl, carbonyl, aromatic, and aliphatic moieties. Functional groups such as hydroxyl and aromatic rings are particularly important for hydrogen bonding and π–π stacking interactions within enzyme active sites, contributing to binding stability [42]. The concordance between FTIR and GC–MS data strengthens the chemical characterization of the extracts and supports the selection of identified compounds for docking studies.

Molecular docking against *C. albicans* N-myristoyltransferase demonstrated that several compounds derived from *A. indica* exhibited favorable binding energies within the catalytic pocket of the enzyme. Among these, Butanedial, bis[(3-hydroxy-4-methoxyphenyl)methylene] showed the strongest predicted binding affinity. The observed binding interactions provide an insight into the antifungal activity previously observed for *A. indica* extracts, consistent with prior reports identifying NMT as a promising antifungal drug target [43]. However, these findings should be considered predictive and require further experimental validation. Although oleic acid exhibited favorable binding affinity in the docking analysis, this result should be interpreted cautiously because such ubiquitous metabolites may not necessarily account for the antifungal activity observed in complex plant extracts.

The docking findings complement our recently published in vitro study [14] demonstrating the antifungal efficacy of *A. indica* extract against azole-resistant *C. albicans* isolates. While MIC assays confirmed phenotypic antifungal activity, the present study advances these findings by providing insight into potential interactions. The comparative nature of this study underscores the importance of chemical diversity and molecular target compatibility in antifungal activity.

Several limitations of this study should be acknowledged. GC–MS analysis involves high injector and column temperatures, which may lead to thermal degradation of certain thermolabile phytochemicals and the formation of artefactual compounds. GC–MS analysis is primarily suitable for the identification of volatile and semi-volatile constituents present in plant extracts. However, polar non-volatile phytochemicals are not readily detectable using GC–MS. Therefore, the present GC–MS analysis primarily reflects the volatile and semi-volatile fraction of the extracts, and it is possible that additional polar phytochemicals remain undetected. Although GC–MS provided valuable qualitative information on the phytochemical composition of the ethanolic extracts, future studies employing bioassay-guided fractionation together with high-performance liquid chromatography (HPLC) coupled with liquid chromatography–mass spectrometry (LC–MS/MS)-based metabolomic approaches could facilitate comprehensive metabolite profiling, accurate quantification, and the identification of low-abundance or thermolabile compounds not readily detected by GC–MS. A limitation of the present study is that the phytochemicals identified through GC–MS were not isolated and purified for individual antifungal susceptibility testing. Future studies involving compound purification followed by standardized in vitro antifungal assays, target-specific inhibition studies, and cellular validation are warranted to confirm the antifungal potential of the identified phytoconstituents. Molecular docking provides predictive insights into protein–ligand interactions but does not account for pharmacokinetic behavior, bioavailability, or metabolic stability in biological systems [42].

The docking findings suggest a potential interaction with the selected *C. albicans* target; however, further validation through enzymatic inhibition assays, target confirmation studies, in vivo testing and cellular antifungal experiments is required to substantiate these computational predictions. Therefore, the docking results should be interpreted as supportive rather than confirmatory. Although *Candida albicans* N-myristoyltransferase is considered a promising antifungal target because of its structural differences from the human homolog, the present study did not evaluate ligand selectivity through comparative docking with human NMT or reference inhibitors. Future studies incorporating comparative docking, molecular dynamics simulations, and experimental toxicity assessments will be essential to evaluate target selectivity and the therapeutic potential of the identified phytoconstituents.

Despite these limitations, the integrated experimental and computational framework employed in this study offers a rational and resource-efficient approach for early-stage antifungal discovery, aligning with current strategies for natural-product-based antifungal lead identification [43,44,45,46]. The phytochemicals identified in the present study reinforce the growing interest in plant-derived compounds as promising leads for antifungal drug discovery. Recent research has shifted beyond the identification of natural products toward structural optimization and rational modification of bioactive molecules to enhance antifungal potency, improve physicochemical properties, and overcome emerging drug resistance. Likewise, plant-derived polyphenols have been shown to possess direct antimicrobial activity while also modulating antimicrobial resistance through multiple mechanisms, including disruption of microbial membranes, inhibition of biofilm formation, and interference with virulence-associated pathways. These advances support the continued investigation of the phytoconstituents identified in the present study as potential scaffolds for the development of novel antifungal agents following further isolation, structural optimization, and biological validation [47].

## 5. Conclusions

This study presents an integrated experimental and computational evaluation of *A. indica* and *F. benghalensis* extracts to elucidate their antifungal potential against *C. albicans*. Qualitative phytochemical screening and spectroscopic analyses confirmed the presence of bioactive secondary metabolites, with *A. indica* demonstrating greater chemical diversity, including phenolics, fatty acids, and saponins, compared with *F. benghalensis*. GC–MS and FTIR analyses provided complementary chemical and functional group characterization, supporting the selection of identified compounds for molecular docking studies.

Molecular docking against *C. albicans* N-myristoyltransferase revealed favorable binding interactions for several phytochemicals derived from *A. indica*, offering a plausible explanation for the antifungal activity previously demonstrated in vitro. In contrast, compounds identified from *F. benghalensis* did not exhibit detectable interactions with the selected molecular target.

Collectively, these findings highlight the importance of integrating phytochemical profiling with target-based computational approaches to gain insight into plant-derived antifungal activity. While the docking results provide preliminary supportive evidence for the antifungal activity of the plant extract, further experimental validation is required to confirm enzyme-level effects and to assess pharmacological relevance. Overall, this study contributes to the rational exploration of sustainable, plant-based antifungal strategies and provides a framework for future investigations aimed at addressing antifungal resistance.

## Figures and Tables

**Table 1:** Qualitative phytochemical screening results of ethanolic plant extracts. (Result in all replicates).

S. No.	Phytochemical	Test	Reagents Used	*A. indica*	*F. benghalensis*
1	Alkaloids	Mayer’s test	Mercuric chloride (1.36 g) and potassium iodide (5 g) in distilled water (100 mL)	+ (Cream precipitate)	+ (Cream precipitate)
2	Carbohydrates	Benedict’s test	Sodium carbonate, sodium citrate, copper(II) sulfate	+ (Yellow–orange color on heating)	+ (Yellow–orange color on heating)
3	Phenolic compounds	Ferric chloride test	Water, ethanol, dilute ferric chloride	+ (Dark green color)	+ (Dark green color)
4	Proteins	Biuret test	Sodium or potassium hydroxide and copper(II) sulfate	− (No purple color)	− (No purple color)
5	Flavonoids	Alkaline reagent test	Dilute sodium hydroxide solution	− (No yellow color)	− (No yellow color)
6	Saponins	Foam test	Distilled water	+ (Persistent foaming)	− (No foaming)
7	Amino acids	Ninhydrin test	Ninhydrin in *n*-butanol	− (No purple color)	− (No purple color)

**Table 2:** Chemical composition of ethanolic leaf extract of *A. indica* identified by GC–MS.

Compound	Molecular Formula	Molecular Weight	Retention Time (min)	Relative Abundance (%)
Oleic acid	C_18_H_34_O_2_	282.46	18.22	34.96
Hexadecanoic acid, 14-methyl-, methyl ester	C_18_H_36_O_2_	284.48	18.38	34.89
Dodecanoic acid, 11-oxo-, methyl ester	C_13_H_24_O_3_	228.33	16.63	16.21
4H-1-Benzopyran-4-one, 7-hydroxy-2-phenyl-	C_15_H_10_O_3_	238.24	17.57	2.54
2,5-Bis(4-hydroxyphenyl)-1,3,4-oxadiazole	C_14_H_10_N_2_O_3_	254.24	17.77	2.54
Octadecanoic acid, 3-oxo-, methyl ester	C_19_H_36_O_3_	312.49	20.20	2.33
But-2-endiamide, N,N′-bis(4-methoxyphenyl)-	C_18_H_20_N_2_O_4_	328.36	21.58	1.90
Phenol derivative	C_23_H_32_O_2_	340.50	23.42	1.27
Butanedial derivative	C_20_H_20_O_6_	356.37	25.92	0.85
Benzene,1-[1-ethylpropyl]-4-propyl-	C_15_H_24_	204.35	14.87	0.61
Benzene, (1-methylenebutyl)-	C_11_H_14_	146.23	11.03	0.23

**Table 3:** Chemical composition of ethanolic aerial root extract of *F. benghalensis* identified by GC–MS.

Compound	Molecular Formula	Molecular Weight (g/mol)	Retention Time (min)	Relative Abundance (%)
Phytol	C_20_H_40_O	296.53	17.02	36.90
2-Pentadecanone, 6,10,14-trimethyl-	C_18_H_36_O	268.48	15.28	24.86
1-Eicosene	C_20_H_40_	280.53	15.92	15.04
1-Docosene	C_22_H_44_	308.59	17.85	10.10
1-Pentacosanol	C_25_H_52_O	368.68	19.68	3.34
1-Tetradecene, 2-decyl-	C_24_H_48_	336.64	22.02	1.67
1,4-Eicosadiene	C_20_H_38_	278.52	14.63	2.40
α-Farnesene	C_15_H_24_	204.35	13.82	4.39
3-Methyl-2-butenoic acid, pentadecyl ester	C_20_H_38_O_2_	310.52	19.38	0.84
3,6-Dimethyl-5-hepten-1-ol acetate	C_11_H_20_O_2_	184.28	13.13	0.33

**Table 4:** Binding affinity of ligands from ethanolic leaf extract of *A. indica* against the protein receptor (PDB ID: 1IYK).

Ligand	Binding Energy (kcal/mol)	Inhibition Constant (µM)
Octadecanoic acid	−7.6	2.69
Hexadecanoic acid	−7.4	3.77
Dodecanoic acid	−7.0	7.39
Butanedial, bis[(3-hydroxy-4-methoxyphenyl)methylene]	−8.7	0.42

## Data Availability

The datasets used and/or analysed during the current study are available from the corresponding author on reasonable request.
